# Bioprospecting of inhibitors of EPEC virulence from metabolites of marine actinobacteria from the Arctic Sea

**DOI:** 10.3389/fmicb.2024.1432475

**Published:** 2024-08-30

**Authors:** Tuomas Pylkkö, Yannik Karl-Heinz Schneider, Teppo Rämä, Jeanette Hammer Andersen, Päivi Tammela

**Affiliations:** ^1^Drug Research Program, Faculty of Pharmacy, University of Helsinki, Helsinki, Finland; ^2^Marbio, Faculty for Fisheries, Biosciences and Economy, UiT—The Arctic University of Norway, Tromsø, Norway

**Keywords:** antivirulence, EPEC, arctic marine microorganisms, bioprospecting, actinobacteria

## Abstract

A considerable number of antibacterial agents are derived from bacterial metabolites. Similarly, numerous known compounds that impede bacterial virulence stem from bacterial metabolites. Enteropathogenic *Escherichia coli* (EPEC) is a notable human pathogen causing intestinal infections, particularly affecting infant mortality in developing regions. These infections are characterized by microvilli effacement and intestinal epithelial lesions linked with aberrant actin polymerization. This study aimed to identify potential antivirulence compounds for EPEC infections among bacterial metabolites harvested from marine actinobacteria (*Kocuria* sp. and *Rhodococcus* spp.) from the Arctic Sea by the application of virulence-based screening assays. Moreover, we demonstrate the suitability of these antivirulence assays to screen actinobacteria extract fractions for the bioassay-guided identification of metabolites. We discovered a compound in the fifth fraction of a *Kocuria* strain that interferes with EPEC-induced actin polymerization without affecting growth. Furthermore, a growth-inhibiting compound was identified in the fifth fraction of a *Rhodococcus* strain. Our findings include the bioassay-guided identification, HPLC-MS-based dereplication, and isolation of a large phospholipid and a likely antimicrobial peptide, demonstrating the usefulness of this approach in screening for compounds capable of inhibiting EPEC virulence.

## 1 Introduction

The antimicrobial crisis is the result of the convergence of two phenomena. To begin with, there have been few pharmaceutical antibiotic breakthroughs in recent decades (World Health Organization, 2022). Second, there are reports of increasingly troublesome cases of antibiotic resistance, globally already contributing to millions of deaths annually (Murray et al., 2022). Historically, researchers have sought antibacterial compounds in natural products, particularly in other microbes (Schneider, 2021). And this has had a high success rate; in fact, soil actinobacteria have produced 80% of all currently licensed antibiotics. However, marine actinobacteria found in the sea, on the seafloor or within the microbiome of marine organisms have received far less attention as possible sources of antibiotics, even more so with respect to virulence-modifying compounds.

Inhibiting bacterial virulence is a well-studied alternative method to the more traditional killing of microorganisms or inhibiting their growth (Zambelloni et al., 2015; Defoirdt, 2016; Buroni and Chiarelli, 2020). In essence, the idea is to inhibit the action of virulence causing molecules using pharmaceutical interventions. In the best-case scenario, the treated pathogens would then remain incapable of causing symptoms, but nevertheless alive, and thus selection pressure for resistance would not form so easily. Furthermore, due to their specificity, such drugs would most likely have fewer adverse effects on normal flora, which are affected adversely by drugs inhibiting bacterial growth or viability in general. Many compounds that to date have been described to be able to prevent bacterial virulence have been discovered from natural sources using phenotypic screening assays (Kimura et al., 2011; Duncan et al., 2014; Wu et al., 2019; Mühlen et al., 2021). These include inhibitors of the expression of virulence molecules, inhibitors of the translocation of effectors, pilicides, and adhesion blockers.

Enteropathogenic *Escherichia coli* (EPEC) is a gram-negative bacterium responsible for a significant portion of diarrheal illnesses and mortality in children under five worldwide (Ochoa and Contreras, 2011). EPEC isolates also display many different forms of antimicrobial resistance, including fluoroquinolone-resistance (Eltai et al., 2020), plasmid-mediated carbapenem and colistin resistance, and extended spectrum betalactamases (Karami et al., 2017; Mahmud et al., 2020). EPEC virulence is caused by it adhering to enterocytes and causing lesions in the intestinal epithelium characterized by the destruction of microvilli, a phenomen called attaching and effacing (A/E) lesions (Kaper et al., 2004). Once adhered to the cell, EPEC employs a type III secretion system (T3SS) to deliver various virulence factors into host cells that use the cell's own actin nucleation machinery to induce pathological changes in the cell (Campellone et al., 2002). Among the secreted factors is the translocated intimin receptor (Tir), which is critical for A/E lesion formation (Cleary, 2004). The receptor's ligand is a protein autotransported by the bacterium to its outer membrane, facilitating intimate attachment to the host cell. Once in place, the phosphorylation of the receptor initiates the recruitment of NCK, N-WASP and the Arp2/3 complex leading to abnormal actin polymerization and actin-rich protrusions on the plasma membrane (pedestals) beneath adherent EPEC (Deborah Chen and Frankel, 2005). Consequently, targeting this process holds promise for the development of antivirulence therapies, and it could be inhibited at various different stages of the pathway, for example, by preventing the contact of Tir and its ligand, intimin by orthostatic inhibition of the receptor, via down regulation of the virulence factors, or inhibition of transport via the T3SS.

As a first step toward discovering antivirulence compounds for EPEC infections, we studied the effects of extracts and fractions from four marine actinobacteria for the ability to decrease Tir-mediated virulence and the following abnormal actin condensation within the cells. Additionally, we sought to evaluate the suitability of these assays for screening bacterial extract fractions with a complex mixture of compounds, including potential pan-assay interference compounds (PAINS). The actinobacteria were isolated from sampling sites near Svalbard in the Arctic Sea and identified as using 16S marker gene sequencing. Next, they were cultured in artificial media, extracted for secondary metabolites, and the extracts fractionated for studying their effects against EPEC caused virulence *in vitro*. Three bioactivity screening methodologies were used for each extract. These included (1) testing for their capacity to inhibit the translocation of Tir, (2) their capacity to prevent actin pedestals, and (3) their capacity to inhibit the growth of EPEC in liquid culture. The recognized active fractions were then studied further to narrow down their possible mechanism of action and to elucidate the chemical structure of the active compounds.

Our aim was to design and validate an isolation and automated screening workflow for use with fractions from microbial cultures and explore the presence of virulence-inhibitory compounds within marine bacterial fractions and their potential application for drug development as the complex nature of extracts and extract fractions may interfere with screens that have been developed and validated using pure chemical compounds only. First due to the complex mixtures potentially containing a high number of “promiscuous binders” or pan assay interfering compounds (PAINS), but also due to the high concentration (10–100 μg extract/fraction per mL) commonly tested in initial screens. This requires (1) assaying methodology suitable for high-throughput screening (2) that can be used with complex fractions, not only pure compounds and (3) methodology to isolate and recognize which constituents are the active ones. We show that this workflow can indeed recognize bioactive compounds in these microbial fractions. In addition, the specific inhibition of enteropathogenic *Escherichia coli* (EPEC) virulence could offer an alternative to conventional antibiotic-based approaches, helping to mitigate the issue of antimicrobial resistance over the long term.

## 2 Materials and methods

### 2.1 Chemicals and reagents

pH_2_O was produced by the in house MilliQ system (Merk, Millipore), methanol (HiperSolv, VWR) and acetone (HiperSolv, VWR), dimethyl sulfoxide (DMSO, VWR) were used if not otherwise indicated.

Modified marine ISP2 medium was produced using 4.0 g glucose (Sigma Aldrich) 4.0 g yeast extract (Sigma), 10.0 g malt extract (Sigma), 300 mL filtered seawater, 700 mL dH_2_O, and a trace element solution 0.2% (v/v). Filtered seawater was produced by the seawater supply of the Norwegian college of fishery science in Tromsø, Norway, by filtration through a 0.22 μm Millidisk^®^ 40 filter-cartridge (Millipore). The trace element solution was prepared by dissolving 10% MgSO_4_ 7 × H_2_O, 0.01% FeSO_4_ 7 × H_2_O, 0.01% ZnSO_4_ 7 × H_2_O, 0.01% CuSO_4_ 5 × H_2_O, and 0.01% CoCl2 6 × H_2_O, in pH_2_O, (w/v). The media was autoclaved at 121°C for 30 min using an autoclave (MLS-3781L, Panasonic).

### 2.2 Bacterial strains, culture, and extraction

For the infection model, the EPEC E2348/69 from Bacteriology reference department (BRD) (UK), was used. The strain was transformed using the plasmid pON.mCherry, Addgene #84821 deposited by Howard Schuman, constitutively expressing mCherry, a fluorescent protein, and grown on LB agar plates or LB broth supplemented with 30 μg mL^−1^ chloramphenicol at 37°C and 200 rpm. For more details (see Pylkkö et al., 2021).

For Tir translocation assays, an EPEC E2348/69 strain containing a beta-lactamase chromosomal fusion in LEE5 (for Tir) under the control of the native promotor, was used (CX2135) (Mills et al., 2008). This strain was kindly provided to us by Ilan Rosenshine from the Hebrew University of Jerusalem. This was cultured on LB agar plates or LB broth supplemented with 50 μg mL^−1^ tetracycline at 37°C and 200 rpm.

The actinobacteria strains were isolated from animals collected in the Arctic Sea in August 2020 (Schneider et al., 2022) (listed in Table 1). For the screening of bioactivity, 2 × 500 mL of the strains were cultivated and extracted as described below. For the isolation of compounds, the strains T091 and T160-02 were cultured in 6 × 500 mL modified marine ISP2 medium for 14 days at 20°C and 140 rpm using a shaking-incubator (Multitron Pro, INFORS HT). For extraction, 40 g of Diaion^®^ HP20 Resin (Merck) was used. The resin was activated by incubation in methanol for 30 min and washed with pH_2_O, for 20 min before the resin was added to the cultures and incubated for 3 days. The resin was separated from the cultures using vacuum filtration and cheese cloth filter (1057, Dansk Hjemmeproduktion). The pooled resin for each strain was extracted two times using 2 × 300 mL of methanol for 45 min of extraction. The extract was separated from the resin using Whatman No.3 filter paper and vacuum filtration. The extract was dried *in vacuo* at 40°C.

**Table 1:** Actinobacteria isolates investigated.

**Strain ID:**	**T091**	**T289**	**T060**	**T160-2**
Animal/origin	*Caulophacus arcticus* (*Porifera*)	*Chlamys islandica* (*Mollusca*)	*Dendrobeania sp*. (*Bryozoa*)	*Tricellaria ternata* (*Bryozoa*)
Actinobacterium	*Kocuria* sp.	*Rhodococcus* sp.	*Rhodococcus* sp.	*Rhodococcus* sp.
Location/depth	77.37491402 °N, 8.268010617 °E/1,400 m	74.78804448 °N, 8.57096443 °E/285 m	75.16057145 °N, 13.71765962 °E/1,500 m	75.16057145 °N, 13.71765962 °E/1,500 m
GenBank accession no.	OP537112.1	OP537141.1	OP537103.1	OP537122.1

More detail on the field sites and methodology used to isolate and culture the strains can be found in Schneider et al. (2022).

The exact contents of the used growth media are listed in Supplementary Table S1.

### 2.3 Preparation of fractions

Crude extracts were fractionated using flash liquid chromatography. The extracts were loaded onto resin (Diaion^®^ HP-20ss, Supelco) by dissolving them in 90% methanol aq. (v/v) and adding resin in a ratio of 1:1.5 (resin/dry extract, w/w). Subsequently, the solution was dried under reduced pressure at 40°C. Flash columns (Biotage^®^ SNAP Ultra, Biotage) were prepared by activating the resin by incubation in methanol for 20 min, washing with ddH_2_O, and loading it into the column ensuring the resin being always covered with water. 6.0 g HP-20ss resin was loaded on one column. The fractionation was performed using a Biotage SP4™ system and a water: methanol gradient from 5–100% methanol over 36 min (6 min 5% B, 6 min 25% B, 6 min 50% B, 6 min 75% B, 12 min 100% B) followed by a methanol: acetone step-gradient (4 min methanol, 12 min acetone). The flow rate was set to 12 mL/min. Twenty-seven eluent fractions of 24 mL each were collected in glass tubes and pooled into six flash fractions in total (1–3 were pooled to fraction 1; 4–6 to fraction 2; 7–9 to fraction 3; 10–12 to fraction 4; 13–15 to fraction 5; 16–27 to fraction 6). An appropriate amount of extract-resin mixture was loaded onto the column after equilibration to 5% methanol aq. (v/v). The flash fractions were dried under reduced pressure at 40°C.

### 2.4 Analysis of fractions using HPLC-HR-MS2

For HPLC-HR-MS2 analysis an Acquity I-class UPLC (Waters) was used coupled to a PDA detector and a Vion IMS QToF (Waters). The HPLC was equipped with a Acquity C-18 UPLC column (1.7 μm, 2.1 × 100 mm) (Waters). The mobile phases consisted of acetonitrile (HiPerSolv, VWR) for mobile phase A and pH_2_O as mobile phase B, both containing 0.1% formic acid (v/v) (33015, Sigma). The gradient was run from 10 to 100% B over 13 min at a flow rate of 0.45 mL/min. Samples were run in ESI+ and ESI- ionization mode. The data was processed and analyzed using UNIFI 1.9.4 (Waters). Exact masses were calculated using ChemCalc (Patiny and Borel, 2013). For dereplication an extract of modified marine ISP2-medium and flash fractions of the extract were prepared and analyzed using the same HPLC-MS2 method in order to exclude media components from consideration. (i) PubChem (Kim et al., 2023) and (ii) Chemspider (Pence and Williams, 2010) where used to identify potential compounds during dereplication by elemental composition search (i + ii) and MS-fragment search (ii, implemented in UNIFI).

### 2.5 Isolation of compounds using RP-HPLC-MS via mass triggered fractionation

For the isolation of compounds from flash fractions preparative reversed phase HPLC was used. Fractionation was triggered by the recorded mass signal throughout the chromatographic separation. The HPLC system consisted of a Waters 600 HPLC-pump with a degasser and flow-splitter, a Waters 515 HPLC-pump as a “make-up” pump, a Waters 3100 Mass detector, a Waters 2996 photo array detector and a Waters 2767 sample manager (all Waters). The system was controlled using MassLynx V4.1 (Waters) software A Sunfire RP-18 preparative column (10 μm, 10 × 250 mm) and XSelect CSH preparative Fluoro-Phenyl column (5 μm, 10 × 250mm) (both Waters) were used as solid phases for the first and second round of purification, respectively. The mobile phases for the gradients were A [pH_2_O with 0.1% (v/v) formic acid] and B [acetonitrile with 0.1% (v/v) formic acid]. The flow rate was set to 6 mL/min. Acetonitrile (Prepsolv^®^, Merck) and formic acid (33015, Sigma) were purchased in appropriate quality, ddH_2_O was produced with the in-house Milli-Q^®^ system. For the MS-detection of the eluting compounds one percent of the flow was split and blended with 80% MeOH in pH_2_O (v/v) acidified with 0.2% formic acid (Sigma) and directed to the ESI-quadrupole-MS. The fractions were collected by mass triggered fraction collection and the respective fractions were reduced to dryness under reduced pressure and by vacuum centrifugation, both at 40°C.

### 2.6 Fluorescent actin stain assay

Screening was performed using a modification of the widely used FAS assay published earlier at various concentrations (Pylkkö et al., 2021). In short, this is an imaging-based infection assay. A cell monolayer of 2 × 10^5^ Caco-2 cells mL^−1^ (ATCC CCL-23) is infected with EPEC E2348/69 emitting fluorescence (mCherry) at a MOI of 1:15. This MOI has been determined to be appropriate for clearly distinquizable EPEC-mCherry microcolonies to form during the infection. Following this, actin is stained with phalloidin and nuclei with Hoechst 33342. For each well five fields of view are collected, and all well-level data are mean aggregates of the data from five fields of view. The data is processed using a custom data reduction pipeline which produces as a readout the proportion of all bacterial microcolonies with actin pedestals. This is achieved by segmentation algorithms and a colocalization analysis, each image is segmented into the microcolonies and features are extracted from the channels of both the bacteria and cells within these segments. The main readout produced by this is the proportion of segmented microcolonies that is associated with actin condensation, although other readouts are collected, such as the number and size of microcolonies. The images were analyzed using the custom scripts on a high-performance computing cluster, Puhti, provided by CSC—IT Center for Science, Finland. The code used for analysis is available at https://github.com/tpylkko/FAS-HCS.

To screen the samples, minimum essential media (MEM) preincubated (1:50, 3 h) mCherry-EPEC suspension at 2 × concentration was added to 96-well source plates (NUNC) using a dispenser (Mantis, Formulatrix), and the fractions dissolved in 2.5% DMSO-MQ were added in 2 × concentrations so that the correct concentration of the samples and bacterial suspension with a multiplicity of infection of 1:15 was achieved in the source plate. A volume of 60 μL of this mixture was transferred to the screening plates (Phenoplate 384, PerkinElmer) and the plates were centrifuged at 1,000 × g for 4 min to allow the bacteria to come into contact with the cells. The plates were subsequently incubated for 2 h at 37°C, 95%, humidity 5% CO_2_ (Biospa, Biotek). After this, a staining solution was applied to the plate using a dispenser (Mantis, Formulatrix) and incubated at RT for 20 min. The plate was then washed three times (100 μL) with Hanks buffered saline solution (HBSS) with an automated liquid handling workstation (Biomek i7, Beckman Coulter) and imaged using a protocol in the imaging plate reader Cytation 5 (Biotek). The contents of this solution are in the Supplementary Table S2. More details about image capture techniques are in Pylkkö et al. (2021).

### 2.7 Tir translocation assay

For translocation assays, 2 × 10^5^ Caco-2 cells mL^−1^ (ATCC CCL-23) were seeded into black 384-well plates with transparent bottom (Phenoplate, PerkinElmer, Germany). Bacterial overnight cultures, grown in 50 μg mL^−1^ tetracycline, were diluted 1:50 into MEM with GlutaMAX (Gibco, Germany) and incubated for 2 h at 37°C 5% CO_2_ in a filter capped 50 mL Falcon tube. Hundred microliter of the bacterial suspension was subsequently added to a source plate (96-well, NUNC) to which samples were serially diluted. These plates were incubated for an additional 1 h. Caco-2 cells were washed once with HBSS. The bacteria-sample suspension (60 μL per well) was then added to the cells, the plates were centrifuged at 1,000 × g for 4 min (Eppendorf Centrifuge 5810R) and incubated for 1.5 h (37°C, 5% CO_2_). Media was then removed, and infected cells were washed twice with 60 μL HBSS. MEM with 100 μgmL^−1^ gentamicin was added to the cells and mixed with LifeBLAzer CCF4-AM staining solution (Invitrogen). The plates were then incubated for 1 h at room temperature. Subsequently, the fluorescence was determined in a Cytation 5 (Biotek, Germany) using an excitation wavelength of 405 nm (10 nm bandwidth). Emission was detected with 460 and 530 nm. Effector translocation was determined by calculating the ratio of blue to green fluorescence (Em520 nm/Em460 nm) following the manufacturer's instructions.

### 2.8 Red blood cell hemolysis assay

EPEC overnight cultures were diluted 1:25 in DMEM high glucose without phenol red (Gibco) and grown for 3 h at 37°C, 5% CO_2_ in the presence or absence of decreasing concentrations of the fractions. Red blood cells (RBC) were purified from Defibrinated Oxoid Sheep Blood (Thermo Fisher Scientific) by three rounds of centrifugation in 1.5 mL eppendorf tubes using a table top centrifuge at 2,000 × g and washing with PBS, then resuspended to 5% (v/v) in DMEM high glucose without phenol red. Bacterial cultures were equalized to 10^8^ in 100 μL and added to 100 μL sheep RBCs (5% v/v) in a 96-well plate. Uninfected RBCs in DMEM were used as a negative control. Total lysis was achieved by adding 0.5% Triton-X to the culture medium. To synchronize infection and mediate bacterial-cell contact, tubes were centrifuged 1 min at 3,220 × g before incubation at 37°C, 5% CO_2_. After 2 h, cells were gently resuspended, followed by centrifugation at 3,220 × g for 1 min. Fifty microliter of each supernatant was transferred to a 96-well plate, and the amount of hemoglobin released was assessed at 543 nm in Cytation 5 (Biotek) plate reader. Hemolysis was calculated as the percentage of hemoglobin released by the DMSO-treated wild-type-infected RBCs.

### 2.9 Analysis of the inhibition of growth of EPEC

Wild-type EPEC E2348/69 was grown overnight in LB broth at 37°C, 200 rpm and resuspended to 2 × 10^6^ cells mL^−1^. For the assay, 100 μL were added to each well of a 384-well plate containing appropriate amounts of sample or gentamicin 4 μg mL^−1^ as a control. Plates were incubated at 37°C, 95% humidity without shaking (Biospa, Biotek) and the OD_600_ was determined every hour for 24 h using Cytation 5 (Biotek).

## 3 Results

**Figure 1:**
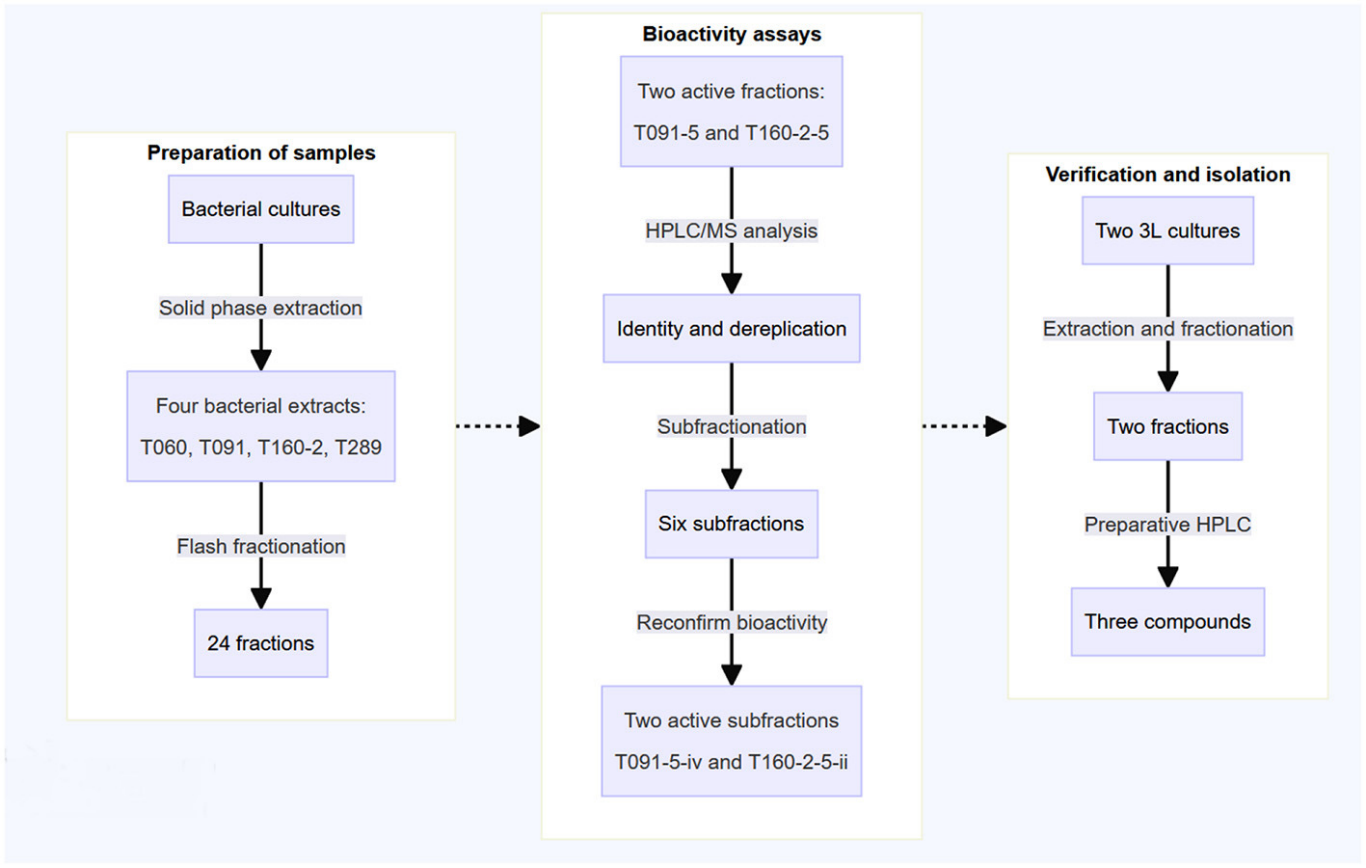
The schematic representation outlines the workflow employed in this study. Previously collected marine actinobacteria were cultured in 500 mL flasks in marine modified ISP2 media. Subsequently, the extracts underwent solid-phase extraction and fractionation using reversed-phase liquid chromatography, resulting in the generation of six fractions per sample (24 in total). The crude fractions were then screened for bioactivity in virulence-related *in vitro* assays, monitoring both the translocation of Tir and actin pedestal formation. The active fractions were also checked for their capacity to induce red blood cell hemolysis. Following this, the two active fractions were further fractionated and the activity of these subfractions was reconfirmed. Finally, the isolation and analysis of the active compounds was performed using RP-HPLC-MS via mass triggered fractionation.

The actinobacteria strains investigated in this study were obtained during a research expedition aboard the Norwegian research vessel Kronprins Haakon in the Arctic Sea. These strains were isolated from samples of invertebrates (Schneider et al., 2022) (see Table 1, Section 2.2) Bacterial strains, cultures and extraction). After collection, the isolates were cultured in marine modified ISP2 media. Subsequently, they were subjected to solid-phase extraction using HP20-resin, followed by fractionation into six fractions via FLASH liquid reversed-phase chromatography. These fractions were then screened using various EPEC virulence related *in vitro* assays and further investigation was conducted on the bioactive fractions to identify the active compounds responsible for these effects (see Figure 1 for overview of workflow).

### 3.1 Fractions 5 from a *Kocuria* sp. and a *Rhodococcus* sp. reduce actin pedestals

To evaluate if the bacterial extract fractions suppress the establishment of actin pedestals, a hallmark of EPEC infections, we employed a high-content screening format of the commonly used fluorescent actin staining (FAS-HCS) assay (Knutton et al., 1989; Pylkkö et al., 2021). When EPEC infects host cells it causes actin-rich pedestals in the vicinity of the microbial microcolonies adhered to the host cells (Campellone et al., 2002). These structures—often called pedestals in the literature—can be visualized using actin staining in cell culture monolayers by phalloidin conjugated fluorophores. This assay additionally quantifies the counts and size of EPEC microcolonies. Previous research in our labs has indicated that these readouts can be used to recognize known antibiotics, as microcolony counts and size tend to show dramatic decrease with these treatments (Pylkkö et al., 2023). Bacteria are typically not entirely killed (and washed away) by short-term treatment with antibiotics, particularly at lower concentrations.

**Figure 2:**
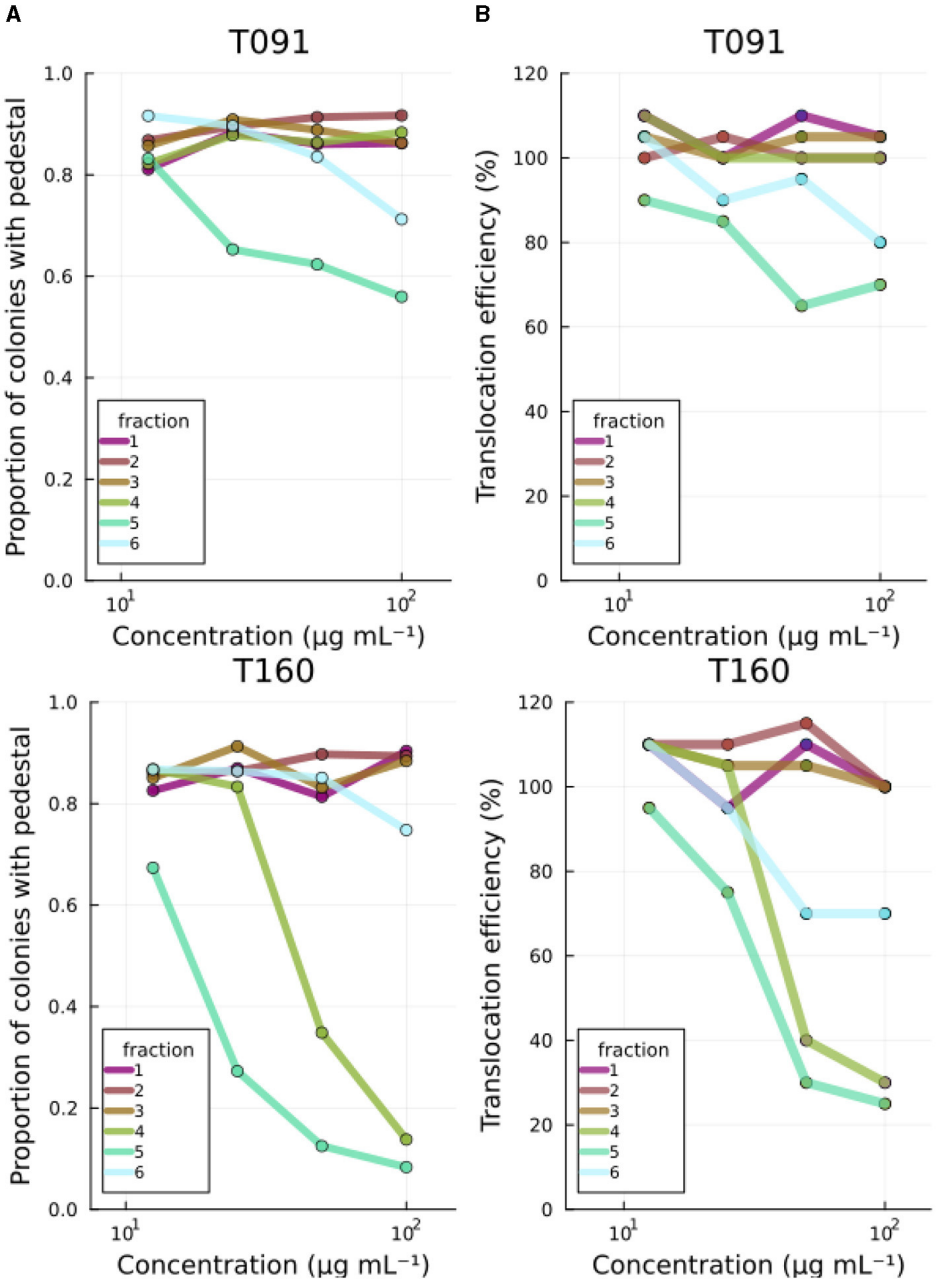
Primary screening results. **(A)** Fractions 5 from T091 and T160-2 decrease both the number of EPEC colonies with actin pedestals and **(B)** the translocation efficiency of the translocated intimin receptor in a concentration-dependent manner. The images are segmented by microcolonies, and the actin condensation under these is analyzed from the equivalent area in the phalloidin channel from the cells by segmenting inside of the first segments from the bacterial signal, thus creating a readout of proportion of microcolonies with detectable actin condensation beneath. For translocation assays, the readout is based on the FRET signal from the LifeBLAzer CCF4-AM dye and normalized to No treatment. The primary screening was performed at four concentrations (100, 50, 25, and 12.5 μg mL^−1^). Fractions from the other strains had no effect (see Supplementary Figure S1).

Fractions originating from two of the bacteria (fractions 5 of T091 *Kocuria* sp. and T160-2/*Rhodococcus* sp.) reduced actin pedestals (Figure 2A). The subsequent fraction (fr.6) also had a similar, but weaker effect, likely due to the fact that they contain the same compounds in lower quantities. The other fractions did not display such activity (Supplementary Figure S1). A reduction in the assay readout can occur due to mechanisms relating to bacterial virulence, such as the constituents inhibiting the adhesion of EPEC to the cells, or down regulating virulence related proteins. However, it can also occur due to other indirect mechanisms such as the compounds being toxic to the organisms, or somehow obscuring the image processing pipeline. Therefore, further studies were conducted.

### 3.2 Fractions 5 from a *Kocuria* sp. and a *Rhodococcus* sp. also reduce Tir translocation

In order to cause pathological changes, such as A/E lesions and associated actin pedestals, EPEC injects virulence factors (e.g., Tir) into the host using a molecular syringe-like device called the type three secretion system. The efficiency of virulence molecule translocation has been studied using beta-lactamase reporter fusions. In this high-throughput method, one C-terminally tags the effector of interest (Tir in this study) with the TEM-1 β-lactamase and infects cells loaded with a FRET signal capable molecule (CCF2-AM) (Mills et al., 2008). CCF2-AM is a molecule in which the donor and acceptor fluorophores are linked together with a beta-lactam. Therefore, the signal emitted by the cells correlates with the degree to which the reporter fusion enzyme (beta beta-lactamase) has cleaved the intracellular molecule, and thus, indirectly with the efficiency of translocation. Beta-lactamase enzymes will not normally be present in unmodified cultured human cell lines. The marine bacterial fractions previously recognized as inhibiting actin pedestals (T091-5 and T160-2-5) also reduced the translocation efficiency of Tir to the infected cells in a concentration dependent manner in this assay (Figure 2B).

### 3.3 Fractions from a *Rhodococcus* sp. T160-2 prevent red blood cell hemolysis

**Figure 3:**
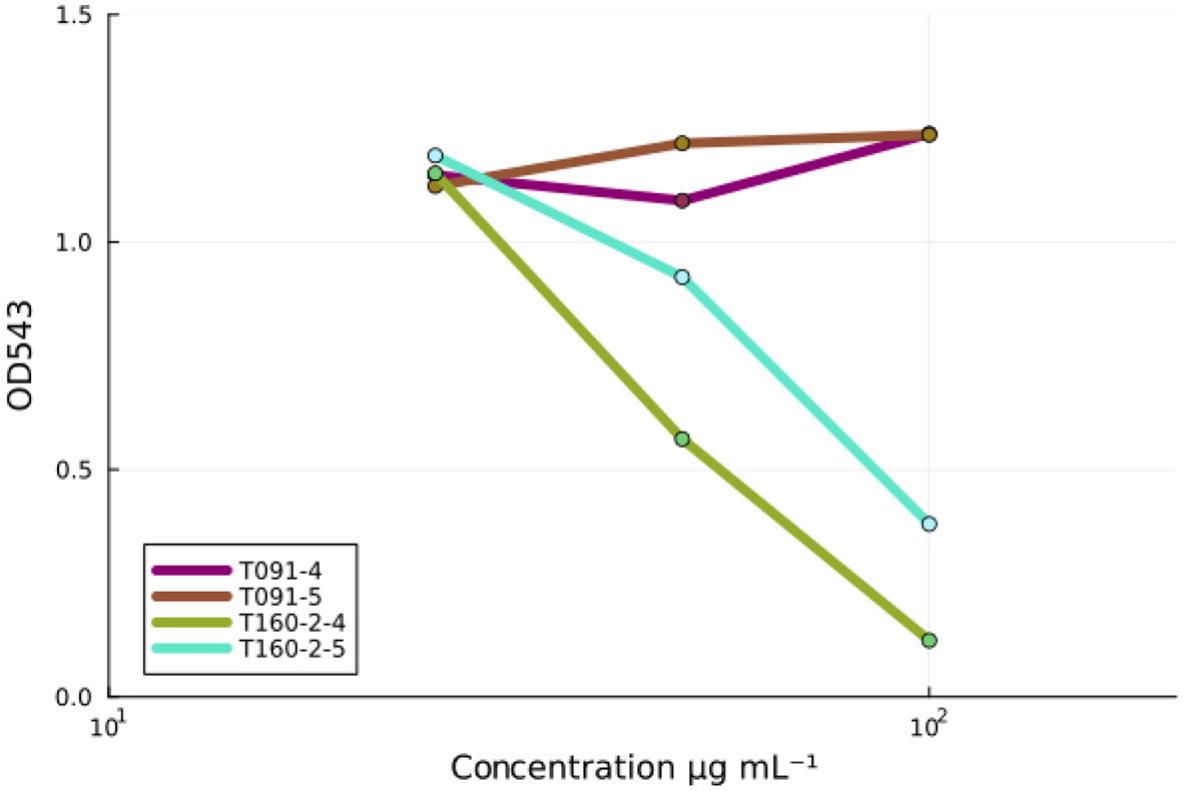
The inhibition of EPEC-induced red blood cell hemolysis by fractions. Fractions (4 and 5) from the strain T091 do not prevent EPEC induced hemolysis of sheep red blood cells at any concentrations, whereas fractions (4 and 5) from the strain T160-2 inhibit red blood cell hemolysis in a concentration dependent manner (100, 50, and 25 μg mL^−1^). Linear models fit to the data: T091 β_fraction4_ = −0.013, *p* = 0.17, β_fraction5_ = −0.011, *p* = 0.003 and T160-2 (β_fraction4_ = 0.00, *p* = 0.46, β_fraction5_ = 0.00, *p* = 0.35). These results suggest that the observed activity in the screening assays is not due to the inhibition of T3SS-based injection, as compounds, such as aurodox, that do inhibit the expression of the T3SS injection needle, are known to inhibit hemolysis.

EPEC infection causes rapid red blood cell hemolysis, and this is likely caused by the injection of EspB and EspD proteins that form a pore on the plasma membrane, which is then used by the type three secretion needle complex to inject molecules into the host cell (Luo and Donnenberg, 2006). Some compounds, such as aurodox for example, can prevent hemolysis efficiently even if they may only modestly prevent actin pedestals (Kimura et al., 2011; Pylkkö et al., 2021). Nevertheless, in *in vivo* murine infection experiments with *Citrobacter rodentium*—a murine specific A/E-pathogen—aurodox protected the entire treatment cohort from death, while all individuals were lost in the no treatment condition and antibiotic treated condition by day 13 (Kimura et al., 2011) suggesting that such compounds may nevertheless be useful. Fractions 4 and 5 from T091 neither showed decrease in hemolysis activity in a dose-dependent manner (β_fraction4_ = 0.00, *p* = 0.46, β_fraction5_ = 0.00, *p* = 0.35) but fractions 4 and 5 from T160-2 did (β_fraction4_ = −0.013, *p* = 0.17, β_fraction5_ = −0.011, *p* = 0.003) (Figure 3).

### 3.4 Fraction 5 from the *Rhodococcus* sp. T160-2 prevents formation of microcolonies and the growth of EPEC

**Figure 4:**
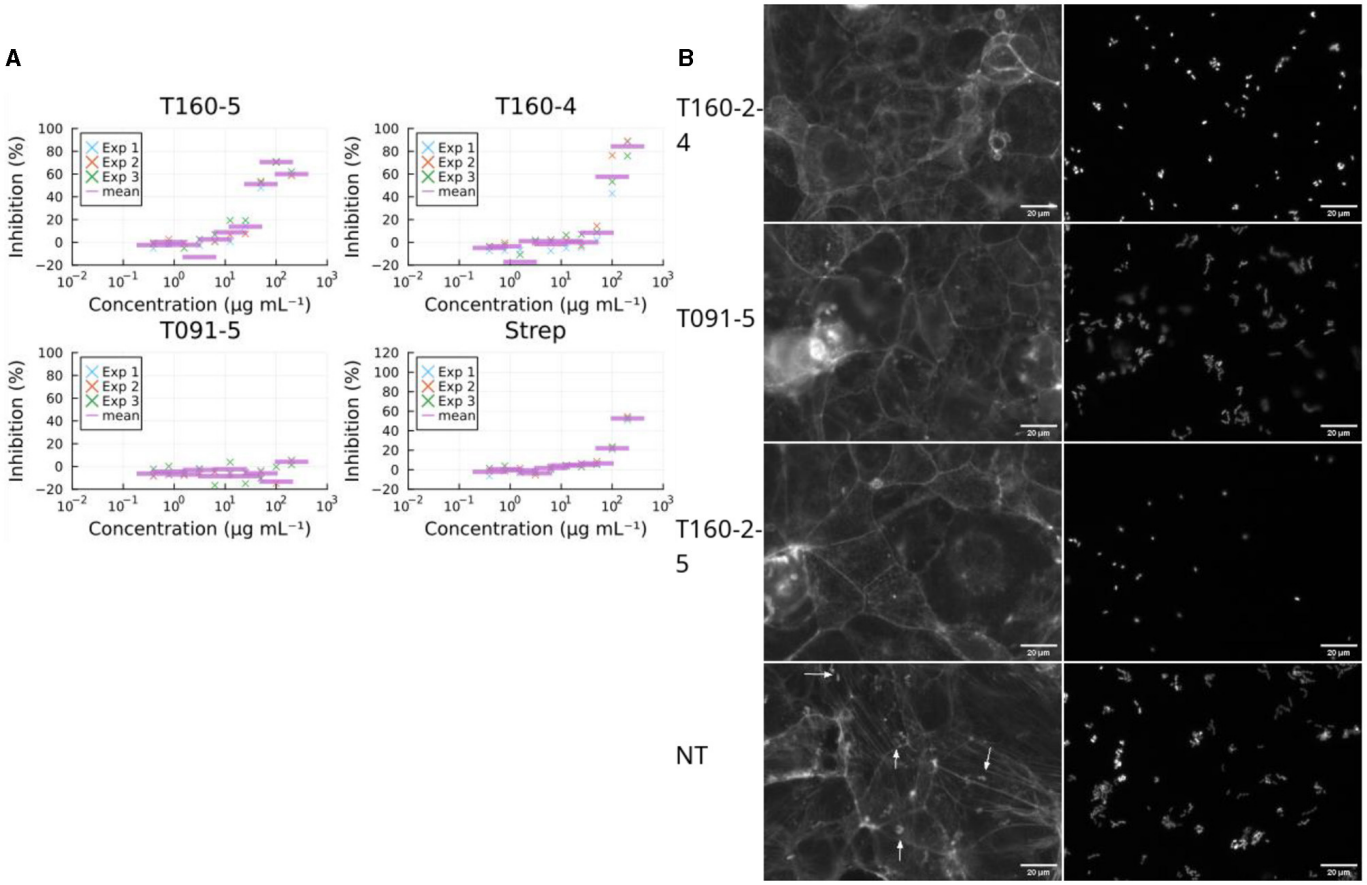
Effects on growth. **(A)** Fraction T091-5 did not inhibit growth in the broth microdilution assay (at 6 h post infection, three biological replicates marked by “×” and their arithmetic mean with a purple bar). This supports the view that the decrease in virulence caused by the compounds in fraction 5 of T091 do not achieve this via a bactericidal or growth inhibiting mechanism of action, whereas the compounds of fractions 4 and 5 from T160-2 are likely growth inhibitory. Streptomycin is shown for comparison, the strain (E2348/69) carries resistance genes for streptomycin (*strA* and *strB*), in contrast T091-5 does not show inhibition at any concentration. The results are normalized to the no treatment condition and 4 μg mL^−1^ gentamicin. **(B)** Fractions also change microcolony morphology in the coinfection model. In the no treatment condition (Size mean = 102) the bacteria form normal microcolonies, where multiple bacteria adhere in a pattern called “localized adherence”, whereas in the growth inhibiting conditions smaller colonies or even individual bacteria are only present. Mean sizes at 100 μg ml^−1^ T160-2 f4 = 54.7, T160-2 f5 = 59.7 and T091 f5 = 137. Arrows indicate actin condensation foci, the scale bar is 20 μm in size.

Bacterial virulence can also be inhibited, via non-specific mechanisms, i.e., generalized toxicity, such as that caused by growth inhibiting antibiotics. Inspection of the images revealed normal looking microcolonies when treated with fraction 5 from T091, suggesting that this was not the case (Figure 4A). Antibiotics typically cause the abolishment of microcolonies in the FAS-HCS assay and then very few adherent bacteria are visible on the cells as individual bacteria (no microcolonies), so this can be used as an indirect measure of toxicity. Fraction 5 from T160-2, in contrast, did decrease the size of microcolonies severely (Figure 4A; Supplementary Figure S2). These images also suggest that fraction T091-5 is not preventing adhesion entirely either, as there are clearly bacteria and microcolonies in the images (Figure 4A). Therefore, all fractions showing activity were also assessed for growth inhibition of EPEC in broth microdilution assays. Fraction 5 from T091 did not inhibit growth in this assay at concentrations up to 100 μg mL^−1^, whereas fraction 5 from T-160-2 did (Figure 4B).

### 3.5 Individual subfractions of the crude FLASH fractions explain the bioactivities

Because the flash fractions contain a several compounds, further subfractionation (refractionation) of these primary fractions was performed using HPLC in order to reconfirm the activities and to get an improved understanding of the specific compounds involved. These subfractions were retested in the assays and then subjected to mass spectrometry to investigate their individual constituents. Following this, the individual molecules of the fractions were purified and tested again in the assays to reconfirm the active ones.

**Figure 5:**
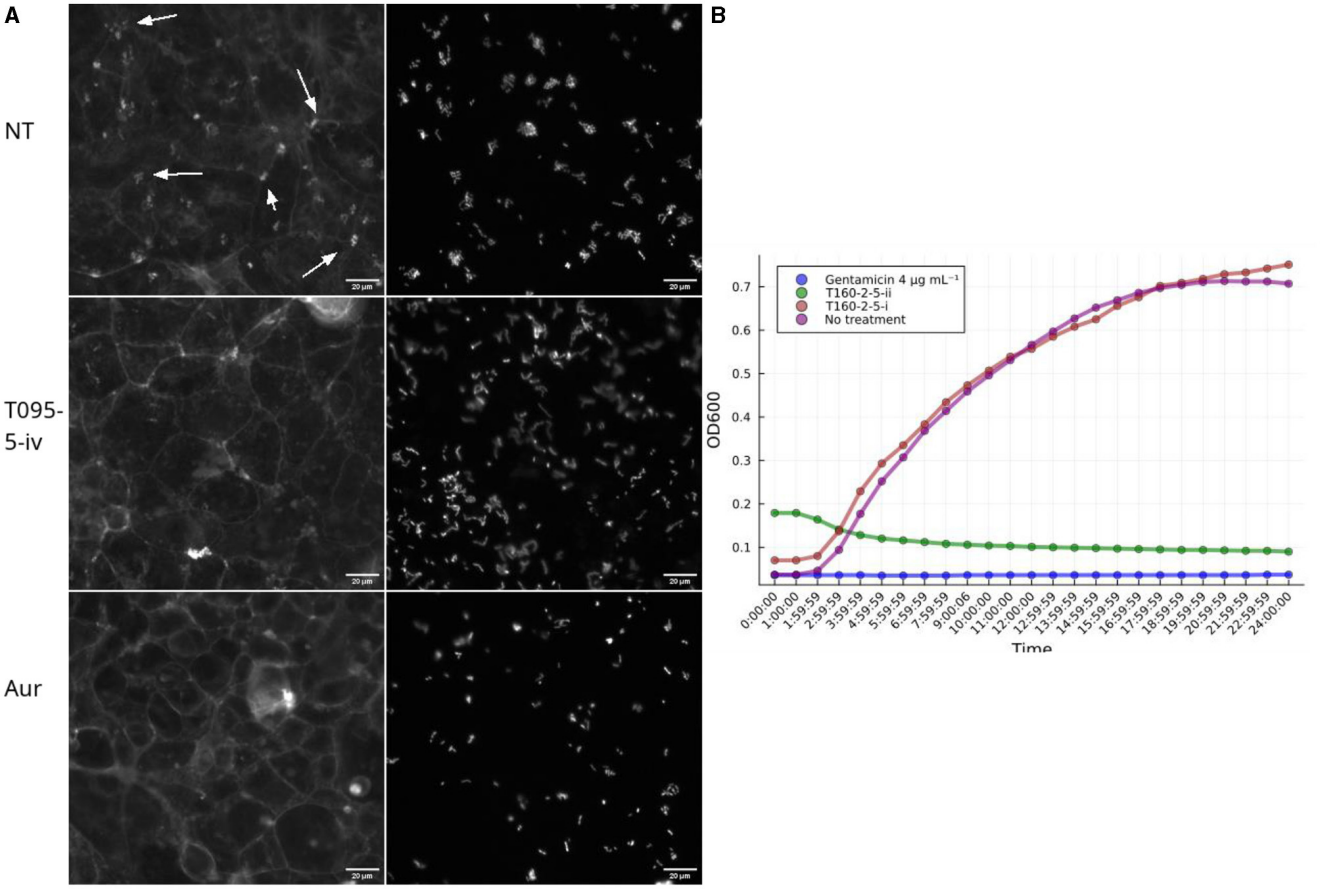
Subfractions demonstrate a similar biological activity as the full fractions in the screening assays. **(A)** Example images of pedestal inhibition. Each picture depicts a Caco-2 cell monolayer that is stained using phalloidin-Alexafluor488 and on the right EPEC-mCherry. Subfraction iv from T091-5 shows decrease in actin condensations, whereas in the no treatment (NT) condition there are multiple pedestals apparent as bright colony-sized spots. Aurodox (aur), a T3SS inhibitor that prevents actin pedestals in EPEC infections. **(B)** The two subfractions (i and ii) isolated from the fifth flash fraction of T160-2 had opposite effects on growth kinetics of EPEC, so that T160-2-5-ii is inhibitory similar to 4 μg mL^−1^ gentamicin, whereas T160-2-i is similar to no treatment.

From T091-5, four subfractions, supposedly representing the major constituents (i, ii, iii, iv) were isolated using MS-coupled preparative HPLC equipped with a RP18 column, and then re-examined in the FAS-assay by dissolving into 2.5% DMSO with MQ, and applied to the cells dissolved in 35 μL of MEM. From these one (iv) decreased the average proportion of colonies with pedestals (0.63) compared to the no treatment (0.84) condition of the entire fraction, whilst the rest seemed to have lesser or no effect. Aurodox had a slightly lower mean effect of 0.56. Inspection of the images indicated similar decreases in actin condensates underneath the colonies between iv and aurodox (Figure 5A). Similarly, fraction T160-2-5 was subfractionated into two subfractions (i and ii), of which one (ii) showed similar growth inhibitory activity, whereas the other one (i) did not show any kind of growth inhibition (Figure 5B).

### 3.6 Isolation of compounds

Two subfractions were therefore considered bioactive. From strain T091 two compounds were isolated. The 3 L culture of T091 yielded 3.68 g of extract that was fractionated into six fractions. Fraction 5 (321 mg) and 6 (108 mg) contained compounds **1** and **2**. In the first round of purification using the setup described under Section 2.5, the two molecules were isolated using a Sunfire RP18 column and a gradient from 25 to 100% (v/v) B in 15 min, the quadrupole was recording m/z from 200 to 800 in ESI+ and the signals m/z 782.5 and m/z 769.5 (low resolution MS) were set to trigger the collection of the eluents. The retention times were 8.36 min for **1** (yielding 2.4 mg) and 8.74 min for **2** (yielding 5.3 mg). In a second round of purification **1** was further purified using a Fluoro-Phenyl column and a gradient from 25 to 100%B (v/v) in 15 min, the retention time of 1 within that condition was 7.19 min. **2** was purified in a second purification step using a Fluoro-Phenyl column and a gradient from 25 to 75% (v/v) B in 14 min, the fraction from the first purification still contained traces of **1**. Compounds **1** and **2** were therefore collected at retention times of 7.87 and 8.25 min, respectively. The isolations were executed by multiple injections and pooling of the respective fractions. The final yields after pooling and drying were **1**: 1.2 mg of red-brown wax-like compound and **2**: 0.9 mg of pale-brown wax-like compound. However, upon attempting to isolate more of the compound, isolation of **3** from 3 L culture of T160, yielding 2.01 g of crude extract was not successful, no visible or weighable amount of compound could be collected.

### 3.7 Identification and de-replication of compounds

As the individual compounds causing the bioactivities were recognized, we succeeded to analyze their structure and perform dereplication. The compound (from T091-5) that appeared to be changing the properties of the microculture and adherence of the bacteria was demonstrated to be a phospholipid. HPLC-HR-IMS-MS analysis revealed two potentially bioactive compounds in the active subfraction of T091, which are potential phospholipid-like compounds according to their elemental composition, and one potentially bioactive compound from the subfraction of T160-2-5 (see Table 2).

**Table 2:** Elemental compositions calculated from ESI-HR-MS2 data.

**Compound**	**Observed *m/z* and observed RT**	**Calcd. *m/z***	**Mass error:**	**Observed CCS**	**Formula:**	**Final yield**
1	782.46051 [M + H]^+^ 7.01 min	782.46082	0.396186 ppm	279.99 Å^2^	C_41_H_68_NO_11_P	1.2 mg
2	796.47660 [M + H]^+^ 7.39 min	796.47647	0.163219 ppm	281.52 Å^2^	C_42_H_70_NO_11_P	0.9 mg
3	316.10369 [M + H]^+^ 3.7 min	316.10458	2.815524 ppm	160.04 Å^2^	C_14_H_13_N_5_O_4_	–

## 4 Discussion

Natural product mixtures derived from sources such as plants, bacteria, or animals, comprise a diverse collection of major and minor compounds, and therefore the analysis of them is a more challenging endeavor than the evaluation of individual pure compounds. The complex composition of these extracts requires prefractionation techniques to reduce complexity (Appleton et al., 2007; Tu et al., 2010) and certain constituents within these extracts, such as fluorophores, chromophores, or compounds harboring pan assay interference (PAINS), can directly influence assay outcomes (Bisson et al., 2016; Bolz et al., 2021). The reduced complexity of flash fractions compared to crude extracts and the possibility to compare active with “neighboring” inactive fractions eases the de-replication of active fractions significantly. In our experience, fractionation increases the relative concentration of potentially active compounds (e.g., in relation to media components or inactive metabolites), which then enables their detection using *in vitro* screening. Additionally, employing multiple distinct assays alongside quantitative and qualitative assessment based on raw images enables us to address these complexities effectively. Imaging and other high-content methods are notably less susceptible to interference on detection signals, as the artifacts can typically be directly recognized from the images by the operator or well-designed quality control in the analysis pipeline. For example, surfactants, particularly rhamnolipids, have recurrently exhibited bioactivity when screening extract fractions from marine bacteria, demonstrating efficacy in both antibacterial and anticancer screenings within our laboratory (Schneider et al., 2019; Kristoffersen et al., 2021). In this study, we noticed very little, if any, interference in the measurements based on optical density, despite some of the fractions being somewhat dark in appearance. It is worth noting that the widely used optical density measuring hemolysis assay has been modified into an imaging-based method, wherein problematic fractions could be reevaluated in case of uncertainty (Knutton et al., 2002). Nonetheless, to our knowledge, this study marks the first utilization of the Tir-translocation and FAS-assays for bioassay-guided discovery from bacterial extract fractions, providing evidence for the applicability of these assays as valuable tools for the bioprospecting of specific inhibitors of virulence.

Many antimicrobial and virulence inhibiting compounds have previously been discovered in natural products of especially microbial origin. For example, one of the first type three secretion system inhibitors ever discovered was the glycolipid caminoside A isolated from extracts of the marine sponge *Caminus sphaeroconia* (Linington et al., 2002). The compound was discovered using an ELISA-based high-throughput assay to screen a large (20,000 compounds) library monitoring virulence protein secretion via the pore forming EPEC secreted translocator EspB (Gauthier et al., 2005). Caminoside A decreased the secretion of the EspB from EPEC culture into the supernatant without effect on the secretion of other proteins nor bacterial growth. Guadinomines, the most potent known T3SS inhibitors, were similarly discovered using EPEC-mediated red blood cell hemolysis to screen natural product extracts (of *Streptomyces* sp. K01-0509) (Iwatsuki et al., 2008). In another study, this same screening assay methodology was used to discover polyketides generated by *Streptomycetes* that appear to particularly inhibit virulence molecule expression in EPEC (Kimura et al., 2011). It was later demonstrated that the most potent of these, aurodox, down-regulates virulence genes on a pathogenicity island (Locus of enterocyte effacement) by affecting an upstream regulator, Ler (McHugh et al., 2018). Recently, EPEC Tir translocation inhibitors were discovered from bacterial metabolite collections utilizing a high-throughput translocation screening assay (Mühlen et al., 2021). The active compounds appear to not affect the expression of EPEC virulence genes, but nevertheless decrease the translocation of effectors into the host cell by as of yet unknown means.

We studied the effects of the marine actinobacterial extracts on EPEC virulence and growth. Fraction 5 from the strain T091 inhibited EPEC caused actin condensation and the translation of the translocated intimin receptor. Analysis of the images and data reduced from them on bacterial counts suggests that subfraction T091-5-iv is not eradicating the bacteria, and the fact that this fraction does not show inhibition of growth suggest that it does not have antibiotic activity. Interestingly, the subfraction neither prevented red blood hemolysis, a phenomenon believed to be caused by the pore forming capacity of translocators such as EspB/EspD which are a part of the T3SS injection needle. Antibiotic compounds typically prevent EPEC-induced hemolysis, as do compounds that down-regulate the genes from the Locus of enterocyte effacement pathogenicity island of EPEC. Such down-regulation could otherwise explain the decreased translocation and subsequent actin condensation. However, the compounds (**1**, **2**) in the subfraction (T091-5-iv) responsible for the activity are large (molecular weight around 700) phospholipids by composition and are unlikely to access intercellular compartments of the bacterial cells. Additionally, it is known that EPEC uses multiple pili and attachment molecules both to adhere to cells and to autoaggregate into microcolonies. Because it is known that adherence of typical EPEC strains—such as the EPEC E2348/69 used here—to cells and other EPEC individuals is largely mediated by a type 4 pilus, called the bundle forming pilus, and that one target of this pilus is cell wall phospholipids (Barnett Foster et al., 1999; Wu et al., 2004), it is possible that the compound acts by competing with the membrane-based ligand, thus decreasing the adherence of the pathogen to the cells. For example, naturally occurring EPEC strains that do not express the bundle forming pilus (BFP), for example due to not carrying the pEAF plasmid, do not display localized adherence, but typically adhere in a diffuse pattern (Rocha et al., 2011). The main target of BFP is believed to be phosphatidylethanolamine, which is the second most abundant phospholipid present in the plasma membrane of eukaryotes (Barnett Foster et al., 1999). On the other hand, one would expect to see smaller microcolonies if this were the case. Further investigation is needed to uncover the exact mechanism of action in more detail.

In addition, we discovered that compound **3** from the subfraction T160-2-5-ii from the *Rhodococcus* T160-2 was able to decrease actin condensation and Tir translocation. This, however, showed clear signs of growth inhibition in EPEC both in the images from the infection models, but also in broth microdilution assays. Therefore, these effects are clearly caused by a decrease in viable organisms and not specific virulence related mechanisms. This compound was tentatively identified to be the cause of this EPEC growth inhibiting effect and is under further investigation.

## Data availability statement

The original contributions presented in the study are included in the article/Supplementary material, further inquiries can be directed to the corresponding author.

## Ethics statement

Ethical approval was not required for the studies on humans in accordance with the local legislation and institutional requirements because only commercially available established cell lines were used. Ethical approval was not required for the studies on animals in accordance with the local legislation and institutional requirements because only commercially available established cell lines were used.

## Author contributions

TP: Conceptualization, Data curation, Investigation, Methodology, Software, Validation, Visualization, Writing – original draft, Writing – review & editing. YS: Investigation, Methodology, Writing – review & editing. TR: Conceptualization, Writing – review & editing. JA: Conceptualization, Writing – review & editing. PT: Conceptualization, Funding acquisition, Supervision, Writing – review & editing.
