# Supplementary material for: Bioprospecting of inhibitors of EPEC virulence from metabolites of marine actinobacteria from the Arctic Sea
Source: Front Microbiol. 2024 Aug 30;15:1432475. doi: 10.3389/fmicb.2024.1432475 (PMC11392781; doi:10.3389/fmicb.2024.1432475)
Supplement: Supplementary file 1 [file Data_Sheet_1.docx]

Supplementary Material

# Screening assay results for the six fractions from T289 and T060


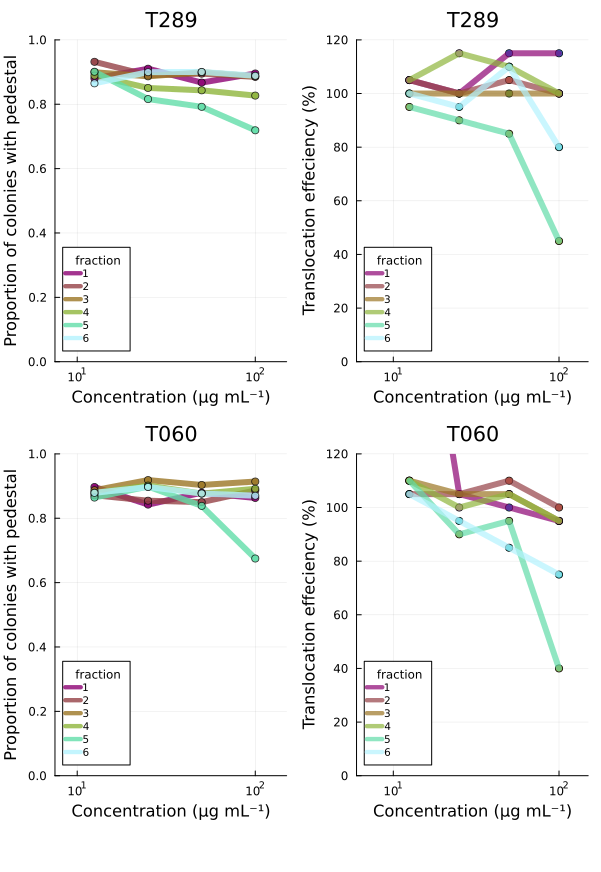


**Supplementary figure 1**. None of the six fractions from T289 or T060 decrease the proportion of colonies with pedestals in a significant manner. The readout from the highest concentrations is lower for fraction 5 and 6, but this effect is only present in a minor form at the highest concentration (100 mg mL^-1^) seemingly not in a dose-dependent fashion.

# Effects of the fractions on colony sizes

**
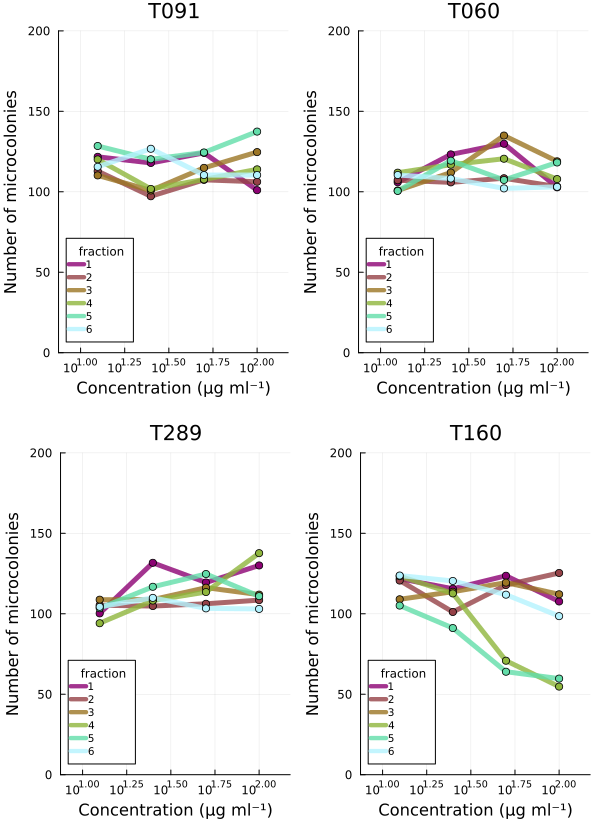
**

**Supplementary figure 2**. Most of the fractions did not have an effect on the sizes of the EPEC microcolonies in the images. However, the growth inhibiting fraction (T160-5) did decrease colonies in a dose dependent manner.

**3 Mass Spectra of Compounds Identified**

Compound **1**


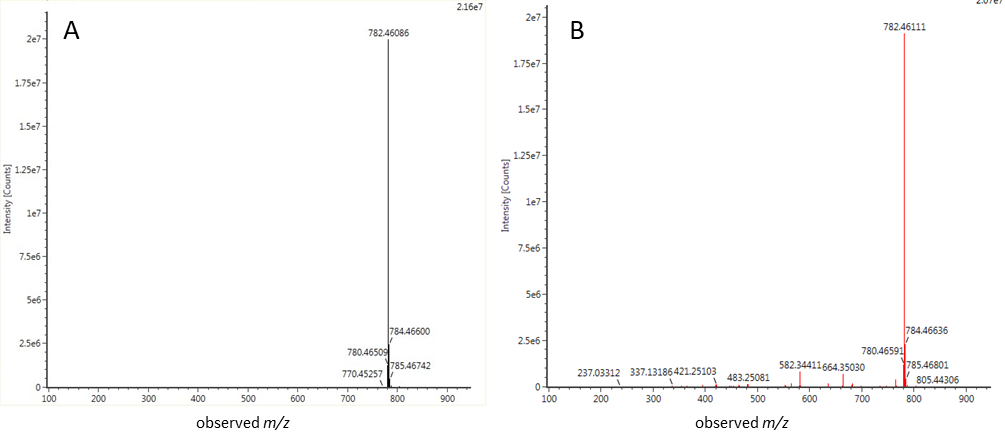


**Supplementary figure 3**. Mass spectra of compound **1**, 5 eV low energy spectra (A) and 20-60 eV (mass dependent ramp) high energy collision/fragment spectra (B).

Compound **2**


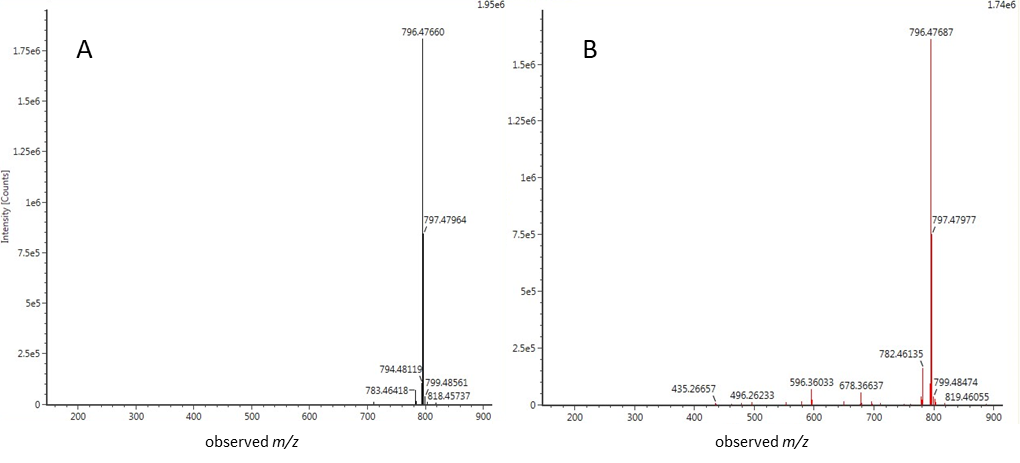


**Supplementary figure 4.** Mass spectra of compound **2**, 5 eV low energy spectra (A) and 20-60 eV (mass dependent ramp) high energy collision/fragment spectra (B).

Compound **3**


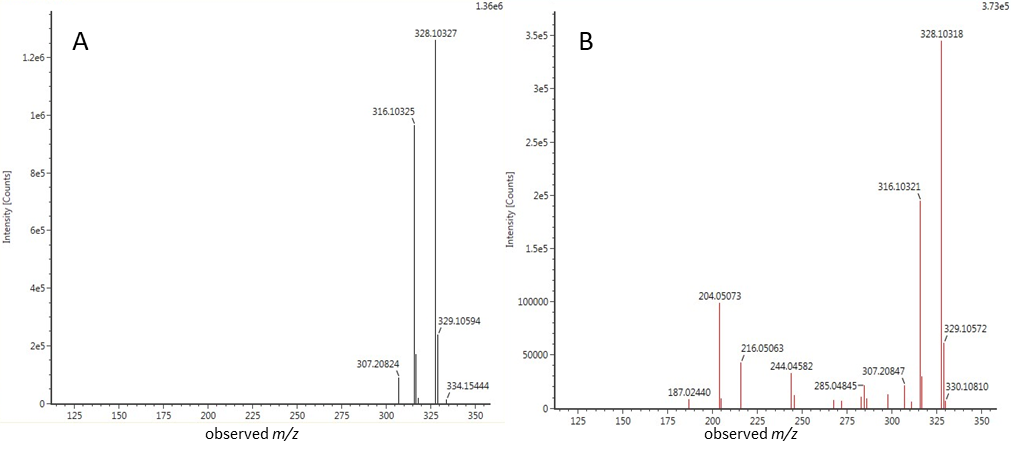


**Supplementary figure 5**. Mass spectra of compound **3**, 5 eV low energy spectra (A) and 20-60 eV (mass dependent ramp) high energy collision/fragment spectra (B). Signal *m/z* = 328.10323 (calc. elemental composition: C_15_H_13_N_5_O_4_ [M+H]^+^; calcd. *m/z* = 328.10458; Mass error: 2.815524 ppm) was observed in HR-MS, the signal was not observable in the preparative HPLC-MS and could therefore not be nominated for isolation.

**Table S1**. Media used for culturing of the microorganisms.

LB media for EPEC

| Reagent | Weight / Volume |
| --- | --- |
| Tryptone | 10.0 g |
| NaCl | 10.0 g |
| Yeast extract | 5.0 g |
| Distilled water | ad 1000 mL |

ISP2-Media with seawater for the culture of the isolates:

| Glucose | 4.0 g |
| --- | --- |
| Yeast extract | 4.0 g |
| Malt extract | 10.0 g |
| Filtered sea water | 300 mL |
| Purified water + 0.2% (v/v) trace element solution | ad 1000 mL |

Table S2. Staining solution for imaging.

| **Reagent** | **Concentration** |
| --- | --- |
| Paraformaldehyde (Media kitchen HiLIFE) | 8% in PBS |
| Phalloidin-AlexaFluor 488 (Invitrogen) | 40 pM (1:250) |
| Triton-X (Thermo Fisher Scientific) | 0.2 % |
| Hoechst 33342 (Thermo Fisher Scientific) | 6 μg/mL |
